# Quantitative MRI Signal Changes of the Spinal Cord as Predictors of Suboptimal Surgical Outcomes in Degenerative Cervical Myelopathy

**DOI:** 10.1111/os.70214

**Published:** 2025-12-12

**Authors:** Xingsheng Zhang, Bingxuan Wu, Tianhua Rong, Bowei Xiao, Ping Li, Baoge Liu

**Affiliations:** ^1^ Department of Orthopaedic Surgery Beijing Tiantan Hospital, Capital Medical University Beijing China; ^2^ Beijing Yanqing District Hospital (Peking University Third Hospital Yanqing Hospital) Beijing China; ^3^ Department of Electrophysiology Beijing Tiantan Hospital, Capital Medical University Beijing China

**Keywords:** degenerative cervical myelopathy, magnetic resonance imaging, prognosis, quantitative analysis, signal intensity

## Abstract

**Objective:**

There are limited data about the association between intramedullary increased signal intensity (ISI) on T2‐weighted magnetic resonance imaging and surgical outcome in degenerative cervical myelopathy (DCM) after anterior decompressive surgery. This study aimed to explore factors contributing to unsatisfactory recovery following surgical treatment for DCM, with a particular focus on evaluating whether preoperative quantitative indicators of ISI on T2‐weighted MRI could be used to forecast surgical outcomes.

**Methods:**

In this retrospective analysis, 94 patients diagnosed with ISI and treated with anterior cervical decompression for DCM between January 2021 and June 2023 were reviewed. Based on a postoperative recovery rate cutoff of 50% at final follow‐up, patients were categorized into optimal and suboptimal recovery groups. Multivariate logistic regression was employed to identify independent predictors of prognosis.

**Results:**

Among the 94 patients, 39 (41.5%) had a suboptimal clinical outcome with a recovery rate below 50%. Multivariate analysis identified longer duration of symptoms, higher signal change ratio (SCR) on T2‐weighted MRI, and the presence of snake‐eye appearance (SEA) as significant predictors of poor recovery. The optimal SCR cutoff value for predicting a suboptimal outcome was 1.53, yielding a sensitivity of 64.1% and a specificity of 83.6%. While somatosensory and motor evoked potentials (SEP/MEP) were associated with baseline neurological function, they did not serve as standalone predictors of recovery.

**Conclusions:**

Longer symptom duration, elevated SCR on T2‐weighted MRI, and SEA features may be significant preoperative indicators of less favorable outcomes in DCM patients. Individuals exhibiting an SCR above 1.53 and SEA on imaging should be considered at increased risk for limited postoperative improvement. These insights highlight the potential benefit of earlier surgical intervention and underscore the need for prospective validation through multicenter studies.

## Graphical abstract

Ninety‐four patients diagnosed with increased signal intensity (ISI) on T2‐weighted MRI and treated with anterior cervical decompression for DCM between January 2021 and June 2023 were reviewed, Higher signal change ratio (SCR), the presence of snake‐eye appearance (SEA), and long symptoms predict poor DCM outcomes, highlighting the need for early surgery.

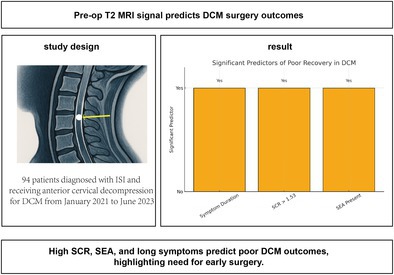

## 1 Introduction

Degenerative cervical myelopathy (DCM) is a prevalent neurological disorder caused by chronic compression of the cervical spinal cord due to degenerative changes associated with aging. This condition often results in gradually worsening neurological impairment and functional decline [1, 2]. At present, surgical decompression remains the gold‐standard treatment to arrest disease progression and enhance neurological recovery. Among the various surgical approaches, anterior cervical surgery is widely accepted for treating DCM, especially in cases involving anterior compression or limited affected levels [1]. Despite appropriate surgical intervention, some patients still experience suboptimal recovery, and the key determinants of surgical outcomes remain controversial [3, 4].

MRI has become an essential diagnostic tool for DCM, providing detailed visualization of spinal cord compression and intramedullary abnormalities suggestive of tissue damage [5]. T2‐weighted increased signal intensity (ISI), first documented by Takahashi et al. [6] has been widely investigated as a potential imaging marker of spinal cord injury. Yet, findings on the predictive value of ISI remain inconclusive, with some studies reporting a negative association with outcomes and others showing no significant link [7, 8, 9]. A possible reason for this inconsistency is the predominantly qualitative nature of ISI assessment in previous research.

To enhance predictive precision, recent investigations have introduced quantitative MRI parameters—such as the signal change ratio (SCR)—to better evaluate ISI severity and its correlation with surgical prognosis [10], In addition, the snake‐eye appearance (SEA) on axial T2WI—a distinct radiologic feature thought to reflect irreversible spinal cord damage—has been identified as a potential poor prognostic indicator in some studies, though its clinical relevance remains under discussion [11].

In addition to imaging, neurophysiological modalities like somatosensory evoked potentials (SEP) and motor evoked potentials (MEP) provide objective measures of spinal cord function. These assessments have demonstrated relationships with baseline neurological deficits, but their capacity to independently predict surgical results remains uncertain [12].

This study aimed to determine key clinical and imaging predictors of suboptimal recovery following anterior cervical surgery for DCM. Special attention was given to quantitative MRI parameters such as SCR, the presence of SEA, and neurophysiological metrics including SEP and MEP. By integrating these diagnostic tools, the goal was to improve early identification of high‐risk patients and guide individualized treatment strategies.

## 2 Methods

### 2.1 Patient

This study received ethics approval from the authors' affiliated institution (Ethics number, IRB: KY2014‐025–02), and informed consent was obtained from all participants to store their data in the hospital database for research purposes. A total of 245 patients who underwent surgery for degenerative cervical myelopathy (DCM) between January 2021 and June 2023 were retrospectively analyzed. Inclusion criteria consisted of patients with increased signal intensity on preoperative T2‐weighted MRI who underwent anterior cervical surgery for DCM. Age from 30 to 80 years and the range of surgical segments is 1–3 levels. The aim of this study is to investigate the efficacy, safety, and prognostic factors of anterior approach surgery for patients with DCM. Anterior approach surgery has unique advantages such as direct decompression and good fusion effect. Exclusion criteria included those with traumatic cervical myelopathy, cervical tumors, cervical infections, previous DCM surgeries, posterior approach surgeries, congenital anomalies, ossification of the posterior longitudinal ligament, and incomplete imaging or clinical data. A total of 94 patients, who were followed up for at least 12 months postoperatively, were included in the final analysis. Surgical procedures were selected based on each patient's condition. Among the patients, 85 underwent anterior cervical discectomy and fusion (ACDF), six had anterior cervical corpectomy and fusion (ACCF), and three received a combined approach of ACCF and ACDF. The operations were performed under a microscope by the same senior surgeon. Through the microscope view, the surgical decompression of the spinal cord was thorough; only when this degree is reached can the operation end.

### 2.2 Clinical Evaluation

Neurological function was evaluated using the Japanese Orthopedic Association (JOA) score [13]; where a score of 17 is considered normal. The recovery rate was calculated using the formula proposed by Hirabayashi et al. [14] Recovery rate (%) = (post‐operative JOA score‐pre‐operative JOA score/17‐pre‐operative JOA score) × 100%. Based on the recovery rate, the patients were divided into two groups: optimal recovery (*n* = 55) and suboptimal recovery (*n* = 39), with a recovery rate threshold of 50%. All patients were treated by the same surgical team.

### 2.3 Radiographic Assessment

Before surgery, all patients underwent neutral lateral and flexion‐extension radiographs of the cervical spine. Additionally, they underwent computed tomography (CT) scans using a 64‐detector scanner (GE Healthcare Bio‐Sciences, Piscataway, NJ, USA) and MRI using a 3.0T scanner (GE Healthcare Bio‐Sciences) at the time of admission. For MRI, sagittal T1‐weighted imaging (T1WI), T2‐weighted imaging (T2WI), and axial T2WI of the cervical cord were obtained using a surface coil. The presence of low signal intensity (LSI) changes on T1WI was also recorded. The Cobb method was employed to measure the cervical lordotic angle between C2 and C7, as well as the cervical range of motion (ROM) during maximal flexion and extension. The Torg–Pavlov ratio was calculated following the method described by Torg JS et al. [15].

**FIGURE 1:**
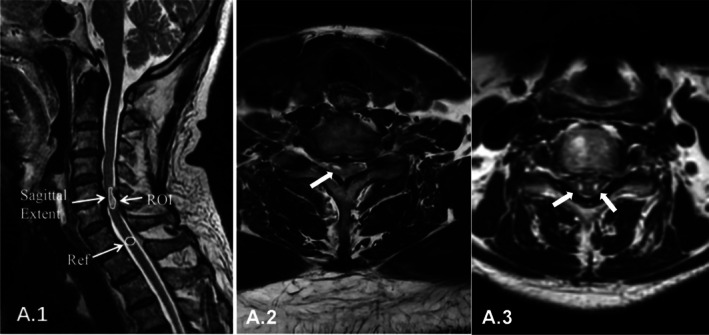
Quantitative measurements of signal change ratio. (A.1) Signal change ratio (SCR) calculation. The region of interest (ROI) represents the circumscribed area of T2‐weighted imaging (T2‐WI) signal change, from which the average intensity values were obtained. Reference values for normal signal intensity in the spinal cord were measured at the C7–T1 disc level, with the area set at 0.3 cm^2^. (A.2) T2‐WI of a patient showing a high‐intensity signal (arrow). (A.3) T2‐WI of a patient demonstrating two nearly symmetrical increased signal intensity spots (arrows), characteristic of the “snake‐eyes” sign on axial T2‐WI.

The increased signal intensity (ISI) values were quantified using ImageJ software (National Institutes of Health, Bethesda, MD, USA), with normal signal intensity values of the cord determined at the C7–T1 disc level. Regions of interest (ROI) were defined at 0.3 cm^2^. The signal change ratio (SCR) was calculated by dividing the ISI value by the signal intensity in the ROI (Figure 1A.1). In cases where two nearly symmetrical ISI spots, referred to as the “snake‐eye” sign, were present on axial T2WI (Figure 1A.3). two orthopedic surgeons independently assessed these measurements, blinded to each other's results and to their own prior assessments. ISI measurements were repeated three times with a two‐week interval, and the average values from both observers were used for analysis.

**FIGURE 2:**
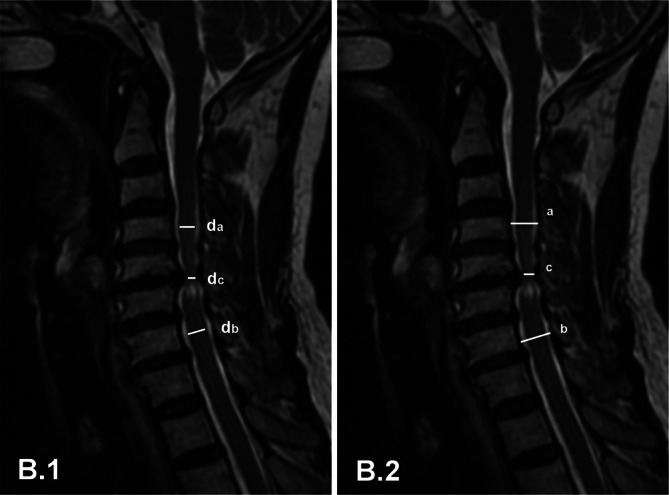
Compression ratio of the spinal cord and canal on sagittal MRI. (B.1) Measurement of the anteroposterior spinal cord diameter (dc) at the most severely compressed region. The diameters (da and db) are measured above and below the first normal vertebral segment, respectively. (B.2) Measurement of the anteroposterior spinal canal diameter (Dc) at the site of most severe stenosis on sagittal MRI. The diameters (Da and Db) are measured above and below the first normal vertebral segment, respectively.

The compression ratio of the spinal cord (CRS) and compression ratio of the cervical spinal canal (CRCS) using the method described by Fehlings et al. [16], was used to assess the severity of spinal cord compression and spinal canal stenosis (Figure 2). The CRS was calculated by comparing the spinal cord diameter at maximum compression on sagittal imaging with the average diameters of the regions above (da) and below (db) the vertebral body. The CRCS was measured similarly to the CRS, as described in a previous study [17]. The sagittal diameter of the spinal cord at the region of maximum compression (dc) was measured, with the imaging plane (midsagittal or parasagittal) selected according to the type of disc herniation (central or paracentral, respectively). Additionally, the uncompressed sagittal diameters at the levels of the superior (ds) and inferior (di) vertebral bodies were obtained.

The CRS and CRCS were calculated as follows:

$$\mathrm{CRS}=\left(1-\frac{\mathrm{dc}}{\left(\mathrm{da}+\mathrm{db}\right)/2}\right)\times 100\;\text{CRCS}=\left(1-\frac{\mathrm{Dc}}{\left(\mathrm{Da}+\mathrm{Db}\right)/2}\right)\times 100$$

### 2.4 Neurophysiological Evaluations

#### 2.4.1 Motor Evoked Potentials (MEP) Examinations

**FIGURE 3:**
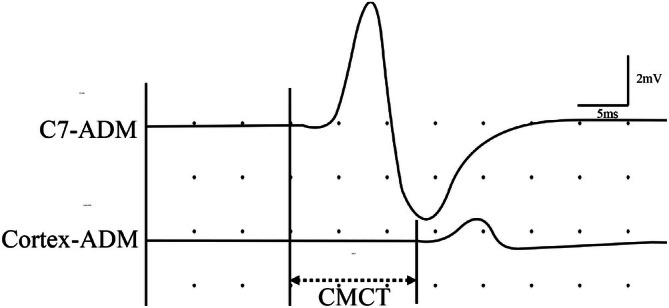
The MEP parameters recorded were total motor conduction time (latency of the MEP after transcranial stimulation at the motor cortex), peripheral motor conduction time (latency of the MEP after transcranial stimulation at the spinous process of the seventh cervical vertebra). The CMCT was calculated by subtracting peripheral motor conduction time from total motor conduction time.

Electrophysiological measurement of the CMCT (Figure 3) was performed to evaluate the clinical severity of the myelopathy in all patients. Muscle responses were recorded using a EDX system (Nicolet Biomedical) with a 3 Hz–10 KHz bandpass filter, while transcranial magnetic stimulation were performed using a 14‐cm‐outer‐diameter round coil and a magnetic stimulator (Model 200, Magstim, Spring Gardens, Whitland, Carmarthenshire, Wales, UK), maximum output magnetic field strength 2.0 T else. An epoch of 50 msec after stimulation was digitized at a 5‐kHz sampling rate. Subject in quiet position, surface recording electrodes were bilaterally placed on the abductor digiti minimi muscles using the standard belly‐tendon method. The reference electrode was placed on the distal tendon at a distance of 3–4 cm from the recording electrode. The sites of stimulation were: the scalp region corresponding to the hand area of the motor cortex, the spinous process of the seventh cervical vertebra (C7) and the elbow point. The stimulation intensity was initially set at 45% and gradually increased until compound muscle action potentials were observed. The cortical stimulation intensity was set at 70%–75% of the maximum output, while that for other regions was maintained at 45%–60%.

For CMCT analysis, data of the worse side were utilized. An MEP response is considered absent if the stimulator output reaches 100% for 10 consecutive trials but no repeatable MEP response occurs. In patients with absent MEPs, CMCT was regarded as 17.5 ms for statistical calculations. This is based on the observation that the maximum CMCT obtained from our patient data was 17.5 ms.

#### 2.4.2 Somatosensory Evoked Potentials (SEP) Examinations

The median nerve was stimulated bilaterally at the wrist at a rate of 3.1 Hz. The stimulus intensity for the median nerve SEPs was 3–4 times the sensory threshold or 1.3–1.5 times the motor threshold to achieve the first contraction of the ipsilateral thumb (thumb flexion of about 1 cm). Recording electrodes were placed at several locations including the Erb's point ipsilateral to the stimulation (EPi), the spinous process of the seventh cervical vertebra (C7S), somatosensory hand cortex at scalp sites C3′ and C4′ (2 cm behind C3 and C4, respectively, Refer to the EEG International 10–20 system), and reference electrodes will be placed at Fz. The evoked potentials were amplified and filtered between 10 and 500 Hz. We averaged 200 responses for median nerve SEPs. Repeat the measurement two or more times and try to make the two curves coincide as well as possible. The SEPs following stimulation on the side with the more severe clinical deficits were used for analysis. The SEP parameters recorded were the following latencies: N9 in the EPi‐Fz lead, N13 in the C7S‐Fz lead and N20 in the C3′/C4′‐Fz lead, and calculated the following intervals: N9–N13, N13–N20, and N9–N20. We analyzed only the following interval: N9–N20 for comparison. An SEP response is considered absent if repeating the above operation three times but no repeatable SEP response occurs. In patients with absent SEPs, N9–N20 was regarded as 14.4 ms for statistical calculations. This is based on the observation that the maximum N9–N20 obtained from our patient data was 14.4 ms.

### 2.5 Statistical Analysis

Continuous variables are presented as mean ± standard deviation, while categorical variables are expressed as percentages. The Student's *t*‐test or Mann–Whitney *U*‐test was used to analyze continuous variables, and Fisher's exact test or chi‐square test was used for categorical variables. Variables with *p* < 0.1 were included in the multivariate logistic regression analysis. Cohen's kappa coefficient was used to measure the agreement between the two observers, and values 0.8–1.0 were considered excellent. To assess measurement reliability, the intraclass correlation coefficient (ICC) was calculated using a two‐way mixed model, with an ICC ≥ 0.75 considered excellent. The area under the curve (AUC) was determined using receiver operating characteristic (ROC) curve analysis to estimate the cut‐off value of pre‐operative SCR as predictors of suboptimal surgical outcomes. An AUC > 0.9 and 0.7–0.9 were considered excellent and good discriminatory performance, respectively. All tests were two‐sided, with *p*‐values < 0.05 considered statistically significant. Statistical analyses were performed using IBM SPSS Statistics (version 25.0, IBM Corp., Armonk, NY, USA).

## 3 Results

### 3.1 Patient Characteristics and Surgical Outcomes

This study involved 94 patients who underwent anterior surgery with DCM, and the mean follow‐up was 15.60 months (range, 12–24 months). The patients comprised 46 men and 48 women with a mean age of 58.0 years (range, 34–78 years). Their average JOA score was 11.8 points (range, 3–15.5) pre‐operatively and 14.7 points (range, 9–17) at the final follow‐up. The mean JOA score recovery rate was 54.8% (range, 7.1%–100%).

### 3.2 Correlation Between Neurophysiological Parameters and Clinical Scores

**FIGURE 4:**
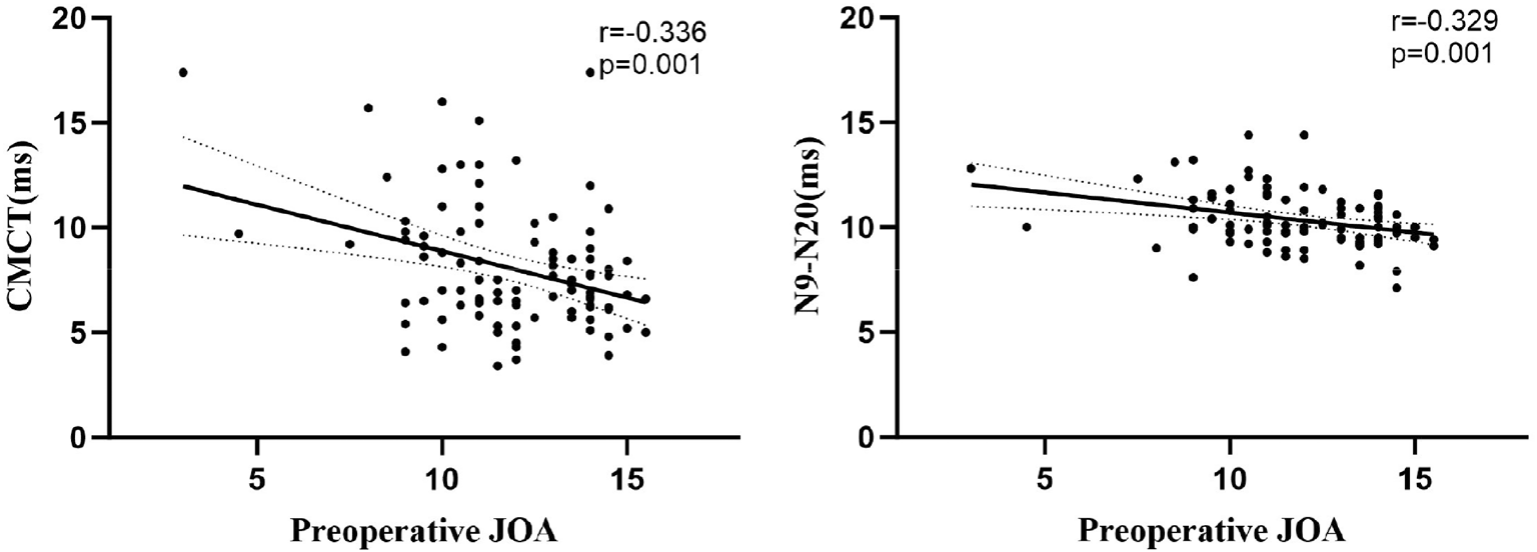
Scatter plot with linear regression line for the CMCT(left diagram) and the interval of N9–N20 (right diagram) versus the preoperative JOA score (*n* = 94).

**FIGURE 5:**
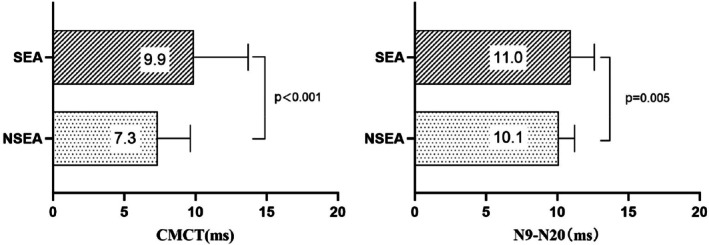
Bar graphs showing the comparison of mean preoperative CMCT (left diagram) and the mean interval of N9–N20 (right diagram) between patients with and without snake‐eye appearance (SEA).

Both upper limb CMCT and N9–N20 interval showed significant negative correlations with preoperative JOA scores (*r* = −0.336, *p* = 0.001 for CMCT; *r* = −0.329, *p* = 0.001 for N9–N20) (Figure 4). Among the 94 patients, 27 patients presented with axial hypersignal as SEA. The CMCT and N9–N20 in the SEA group was significantly prolonged compared with that in the NSEA group, and the difference between the groups was statistically significant (Figure 5).

### 3.3 Predictors of Surgical Outcome

No statistically significant differences in age, gender, hypertension, diabetes mellitus, pre‐operative JOA score and levels involved were found between the optimal group and suboptimal group (*p* > 0.05), which was comparable. Low intensity signal changes on T1WI were observed in 8 (8.5%) of the 94 patients. The pre‐operative C2–C7 lordotic angle, C2–7 ROM, Pavlov ratio, CRS and CRCS were similar in the two groups. The incidence of LSI changes on T1WI was higher in the suboptimal recovery group than in the optimal recovery group; however, there was no statistical difference (15.4% vs. 3.6%, *p* = 0.102).

Compared with patients with optimal recovery, the 39 patients with suboptimal recovery had a longer preoperative duration of symptoms (14.1 vs. 8.2 months, *p* = 0.024). The preoperative SCR was significantly higher in the suboptimal recovery group than in the optimal recovery group (1.61 vs. 1.41, *p* < 0.001). The incidence of SEA changes on the axial plane was higher in the suboptimal recovery group than in the optimal recovery group (46.2% vs. 16.4%, *p* = 0.002). The N9–N20 interval was higher in the suboptimal recovery group than in the optimal recovery group (10.67 vs. 10.12, *p* = 0.047). No statistically significant differences in CMCT was found between the optimal group and suboptimal group (*p* > 0.05), which was comparable (Table 1). Inter‐observer agreement for the detection of signal intensity changes on T1WI and T2WI was very high. The Kappa value was 0.85 for detection of low signal intensity (LSI) changes on T1WI and 0.95 for detection of “snake‐eye” sign present on axial T2WI, respectively. The measurements of SCR showed excellent intra‐rater agreement (ICC = 0.92, 0.90–0.95, *p* < 0.001) and excellent inter‐rater agreement (ICC = 0.91, 0.87–0.94, *p* < 0.001).

**TABLE 1:** Patient characteristics and operative data (*n* = 94).

Variable	Optimal (*n* = 55)	Suboptimal (*n* = 39)	*t/χ* ^*2*^ value	*p*
Age (years)	57.09 ± 9.02	59.28 ± 10.30	1.094^a^	0.277
Gender (*n* [%])			0.643^b^	0.530
Male	25 (45.5%)	21 (53.8%)		
Female	30 (54.5%)	18 (46.2%)		
Symptom duration (mon)	8.15 ± 10.95	14.09 ± 13.25	2.300^a^	0.024^c^
Hypertension (*n* [%])	14 (25.5%)	12 (30.8%)	0.322^b^	0.643
Diabetes mellitus (*n* [%])	5 (9.1%)	4 (10.3%)	0.036^b^	1.000
Pre‐operative JOA score	11.99 ± 2.25	11.60 ± 2.37	0.807^a^	0.422
JOA score at last follow‐up	15.36 ± 1.08	13.68 ± 1.41	6.531^a^	< 0.001^c^
Recovery rate (%)	67.74 ± 13.78	36.55 ± 10.36	11.939^a^	< 0.001^c^
C2‐7 angle (°)	9.02 ± 11.55	10.23 ± 11.91	0.495^a^	0.622
C2‐7 ROM (°)	35.62 ± 10.53	35.85 ± 8.68	0.111^a^	0.912
Pavlov ratio	73.98 ± 9.75	72.93 ± 7.37	0.568^a^	0.571
CRS	29.68 ± 17.53	30.47 ± 17.97	0.214^a^	0.831
CRCS	46.00 ± 16.08	48.39 ± 14.78	0.733^a^	0.465
LSI on T1WI MRI (*n* [%])			2.677^b^	0.102
Yes	2 (3.6%)	6 (15.4%)		
No	53 (96.4%)	33 (84.6%)		
SCR	1.41 ± 0.18	1.61 ± 0.28	3.893^a^	< 0.001^c^
SEA			9.892^b^	0.002^c^
Yes	9 (16.4%)	18 (46.2%)		
No	46 (83.6%)	21 (53.8%)		
CMCT (ms)	7.92 ± 2.71	8.28 ± 3.43	0.564^a^	0.574
N9–N20 (ms)	10.12 ± 1.37	10.67 ± 1.21	2.009^a^	0.047^c^
Levels involved	2.15 ± 0.80	2.21 ± 0.61	0.390^a^	0.698

Abbreviations: CMCT, central motor conduction time; CRCS, compression ratio of the cervical spinal canal; CRS, compression ratio of the spinal cord; ISI, increased signal intensity; JOA, Japanese Orthopedic Association; LSI, low intensity signal; MRI, magnetic resonance imaging; ROM, range of motion; SCR, signal change ratio; SEA, snake‐eye appearance; T1WI, T1‐weighted imaging.

^a^ *t*‐test; results are shown as mean ± standard deviation.

^b^ Chi‐square test; results are shown as *n* (%).

^c^ Indicates statistical significance.

The univariate analysis for the patient characteristics and operative data was conducted. There was no statistical difference between the two groups in the variables of age, gender, hypertension, diabetes mellitus, pre‐operative JOA score, C2–7 angle (°), C2–7 ROM (°), Pavlov ratio, CRS, CRCS, LSI on T1WI MRI, CMCT (ms) and levels involved. The statistical difference between the two groups was analyzed in the variables of symptom duration (months), SCR, SEA and N9–N20 (ms).

### 3.4 Binary Logistic Regression Analysis and Diagnostic Performance

At the binary logistic regression analysis, duration of symptoms, SCR, SEA on the axial plane and N9–N20 interval were eligible for further analysis. The results showed that a suboptimal clinical outcome was associated with a longer duration of symptoms (OR, 1.754; 95% CI, 1.118–2.750; *p* = 0.014), a higher SCR (OR, 4.797; 95% CI, 1.884–12.211; *p* = 0.001) and SEA on the axial plane (OR, 3.197; 95% CI, 1.067–9.584; *p* = 0.038) (Table 2).

**TABLE 2:** Risk factors for suboptimal postoperative outcomes: multivariate logistic regression analysis.

Variable	OR (95% CI)	*p*
Symptom duration (months)^a^	1.754 (1.118–2.750)	0.014^c^
SCR^b^	4.797 (1.884–12.211)	0.001^c^
SEA (*n* [%])	3.197 (1.067–9.584)	0.038^c^
Yes		
No	1.0 (reference)	
N9–N20 (ms)	1.116 (0.762–1.636)	0.573

Abbreviations: CI, confidence interval; OR, odds ratio; SCR, signal change ratio; SEA, snake‐eye appearance.

^a^ Symptom duration (months): (1) < 6 months; (2) ≥ 6 but < 12 months; (3) ≥ 12 but < 24 months; (4) ≥ 24 months.

^b^ SCR: (1) > 1.00 but ≤ 1.50; (2) > 1.50 but ≤ 2.00; (3) > 2.00.

^c^ Indicates statistical significance.

**FIGURE 6:**
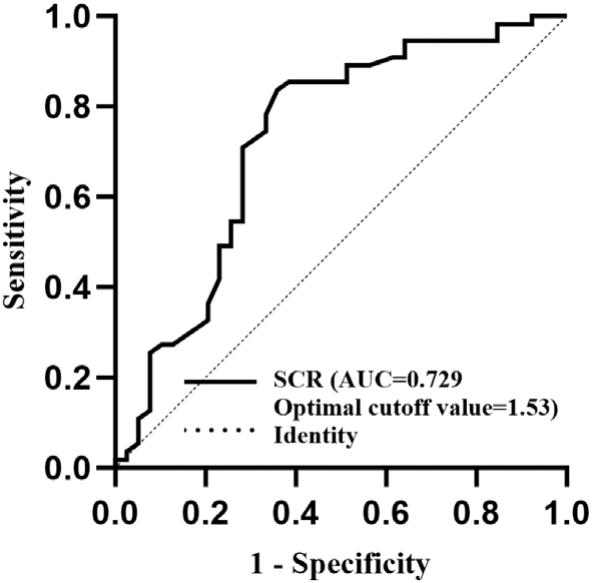
Receiver operating characteristic curve of area ratio of increased signal intensity (ISI) and signal change ratio (SCR). The receiver operating characteristic area is expressed as the area under the curve (AUC).

The ROC curve analysis showed that the cut‐off value of the SCR for predicting a suboptimal surgical outcome based on the Youden index was 1.53 (64.1% sensitivity and 83.6% specificity). The AUC of the preoperative SCR was 0.729 (95% CI, 0.619–0.839; *p* < 0.001). The AUC indicated a good ability of the SCR to discriminate the JOA score recovery rate (≥ 50% vs. < 50%) (Figure 6).

## 4 Discussion

### 4.1 Main Findings of This Study

This study demonstrates that anterior surgical treatment for degenerative cervical myelopathy with intramedullary high signal intensity involving up to three segments can achieve favorable surgical outcomes, with MRI T2 signal intensity serving as predictive factors for postoperative patient prognosis. Three principal findings emerge: (1) Neuroelectrophysiological parameters are closely related to the preoperative spinal cord neurological function status in patients with degenerative cervical myelopathy. (2) Patients with two nearly symmetrical ISI spots, referred to as the “snake‐eye” sign, on axial T2WI have longer SEP and MEP latencies compared to those without the “snake‐eye” sign. (3) Patients with SCR > 1.53 and SEA have poor postoperative prognosis, validating its potential as a predictor of the surgical outcome in patients with CSM.

### 4.2 Anterior Approach Surgical Outcomes in DCM

Recent studies suggest that surgical treatment is considered a good choice for DCM [18, 19]. The surgical fixation methods include anterior, posterior, and combined approaches. Several factors play a role in determining the surgical approach, such as patient age, the direction of spinal cord compression, the segments involved in the surgery, the presence of cervical spine instability, and the sagittal alignment of the spine [20]. Importantly, the general conditions, patient age and baseline myelopathy severity, which are very well documented to be associated with poor recovery after surgery for DCM and are significant confounding factors, are different between the anterior and posterior groups [21, 22]. In our study, patients who underwent anterior cervical approach surgery for DCM were included, and all the patients were limited to a maximum of three levels involved. The mean preoperative JOA score was 11.8 which improved to 14.7 at the last follow‐up. These results were comparable to those reported by He et al. [23], who reported an improvement in JOA scores from 9.8 to 14.9 in 34 patients who underwent the anterior approach. In the current study, the recovery rate was 54.8% which was slightly lower than that reported by Zhang et al. (65.88%) [24]. This is probably because a majority of the patients in this study had moderate‐to‐severe disability preoperatively (moderate‐to‐severe 72.3%, mild 27.7%), as well as variations in follow‐up duration.

### 4.3 Signal Intensity Changes and Axial Lesion Features in DCM Prognosis

The effect of signal intensity changes on patient prognosis has been widely studied. Most investigators believe that post‐operative neurologic functions are associated with pre‐operative ISI changes on T2WI changes [25, 26], whereas other scholars hold the opposite view [9, 27]. Of the 94 patients with the presence of ISI on preoperative T2‐MRI in our study, 39 (41.5%) had suboptimal clinical outcomes with a recovery rate of less than 50%. Therefore, we pay more attention to investigating which MRI findings in patients are correlated with the postoperative outcome. In our study, the signal change ratio (SCR) was used to quantify the degree of hyperintensity. The cut‐off value of the SCR for predicting a suboptimal surgical outcome based on the Youden index was 1.53 which was consistent with previous studies by Wei et al. (1.56) [10], and Zhang et al. (1.46) [28]. Patients with higher SCR values were more likely to have poor recovery outcomes, suggesting that SCR may reflect the extent of irreversible spinal cord injury.

Few studies have focused on the relationships between cross‐sectional area changes of the increased signal intensity and the prognosis in patients with DCM. One related study found that a snake‐eye appearance (SEA) on axial T2WI was an unfavorable prognostic factor for the recovery of upper extremity motor weakness [29]. Another related study found that patients with ISI in both the white and gray matter, based on the location of ISI on sagittal combined with axial MRI, had worse outcomes than those with ISI in the gray matter alone [30]. In our analysis, binary logistic regression confirmed that preoperative MRI findings—specifically, the specific imaging abnormality (SEA) on axial T2‐weighted imaging—are associated with unfavorable prognostic outcomes. Furthermore, results indicated that patients with SEA exhibited significantly prolonged latencies in somatosensory evoked potentials (SEP) and motor evoked potentials (MEP) compared to those without these imaging features. This observation provides a plausible electrophysiological explanation for the poorer clinical outcomes, suggesting that SEA may reflect more severe spinal cord pathology.

### 4.4 Neurophysiological Assessments as Diagnostic Tools Without Prognostic Value

Previous studies have indicated that neurophysiological evaluations, including somatosensory evoked potentials (SEP) and motor evoked potentials (MEP), serve as valuable tools for assessing spinal cord function. However, their utility in predicting postoperative neurological recovery remains a subject of debate [31, 32]. In the present study, we observed that specific neurophysiological parameters—namely, central motor conduction time (CMCT) in the upper limb and the N9–N20 interval—exhibited a significant correlation with preoperative neurological status, thereby reflecting the extent of spinal cord impairment. Despite this association, our analyses revealed that these metrics did not possess substantial predictive capacity for postoperative recovery outcomes. Therefore, neurophysiological monitoring may be more appropriate as a diagnostic tool rather than a prognostic indicator. Future studies should aim to identify more robust predictive biomarkers and validate these observations across heterogeneous patient cohorts to enhance clinical applicability.

### 4.5 T1WI Hypointensity and Recovery Prognosis

The incidence of low intensity signal (LSI) changes on T1WI was 1.9%–55.5% reported in the literature [33, 34]. In this study, hypointense signal changes on T1WI were observed in 8 (8.5%) of the 94 patients. Previous studies have indicated that such preoperative hypointense signal changes may influence surgical outcomes and are associated with a poorer prognosis [9]. In our investigation, the incidence of LSI changes on T1WI was higher in the suboptimal recovery group than in the optimal recovery group; however, the difference did not reach a significant level (15.4% vs. 3.6%, *p* = 0.102). A drawback of this imaging feature is that it presented a low incidence of findings, eight which could lead to a lack of sensitivity. We should be aware that patients with LSI changes on T1WI may translate to a poor neurological recovery, emphasizing the importance of timely surgery.

### 4.6 Strengths and Limitations of This Study

The strengths of the study design include a comprehensive multimodal approach and rigorous validation of predictive factors, enhancing prognostic accuracy in degenerative cervical myelopathy. Several limitations of our study should be considered. First, the study was a single‐center study with a small sample size. Then, the degree of surgical decompression could be evaluated after surgery in our further study to analyze the effect of decompression degree on the neurological symptoms. Furthermore, the only outcome measure used is the JOA score which does not measure the patient's pain and the disability thereof. In addition, this was a retrospective study and therefore had some data bias. Results from this study need to be confirmed by prospective, large‐scale, multicenter studies.

## 5 Conclusions

Preoperative SCR on T2‐weighted MRI, presence of SEA, and longer symptom duration may serve as useful predictors of surgical outcomes in DCM. Patients with SCR > 1.53 and SEA should be considered at higher risk for suboptimal recovery. These findings emphasize the importance of timely surgical intervention and warrant further validation in multicenter prospective studies.

## Author Contributions

**Xingsheng Zhang:** conceptualization, data curation, formal analysis and original draft. **Bingxuan Wu:** writing – review and editing. **Tianhua Rong:** writing – review and editing. **Bowei Xiao:** data curation and original draft. **Ping Li:** data curation and original draft. **Baoge Liu:** conceptualization, methodology, investigation, funding acquisition, writing – review and editing.

## Funding

This work was supported by the National Natural Science Foundation of China (82272524) and the High Level Public Health Technology Talent Construction Project of Beijing (Leading Talent‐02‐05).

## Ethics Statement

The study was approved by the Institutional Review Board. This retrospective chart review study involving human participants was in accordance with the ethical standards of the institutional and national research committee and with the 1964 Helsinki Declaration and its later amendments or comparable ethical standards. The Human Investigation Committee (IRB) of Beijing Tiantan Hospital Affiliated to Capital Medical University approved this study. Ethics number, IRB: KY2014‐025‐02.

## Consent

Informed consent was obtained from all participants.

## Conflicts of Interest

The authors declare no conflicts of interest.

## Data Availability

The data that support the findings of this study are available from the corresponding author upon reasonable request.
